# Current Approaches in NSCLC Targeting K-RAS and EGFR

**DOI:** 10.3390/ijms20225701

**Published:** 2019-11-14

**Authors:** Veronica Aran, Jasminka Omerovic

**Affiliations:** 1 Research Division, National Institute of Traumatology and Orthopedics, Av. Brasil 500, 20940-070 Rio de Janeiro, Brazil; 2 School of Medicine, University of Split, Šoltanska 2, 21000 Split, Croatia; jasminka.omerovic@mefst.hr

**Keywords:** lung cancer, NSCLC, K-RAS, EGFR, lung cancer therapy

## Abstract

The research and treatment of non-small cell lung cancer (NSCLC) have achieved some important advances in recent years. Nonetheless, the overall survival rates for NSCLC remain low, indicating the importance to effectively develop new therapies and improve current approaches. The understanding of the function of different biomarkers involved in NSCLC progression, survival and response to therapy are important for the development of early detection tools and treatment options. Epidermal growth factor receptor (EGFR) and Kirsten rat sarcoma viral oncogene homolog (K-RAS) are two of the main significant biomarkers for the management of NSCLC. Mutations in these genes were associated with development and response to therapies. For example, the use of small molecule tyrosine kinase (TK) inhibitors and immunotherapy has led to benefits in some, but not all patients with altered EGFR. In contrast, there is still no effective approved drug to act upon patients harbouring K-RAS mutations. In addition, K-RAS mutations have been associated with lack of activity of TK inhibitors. However, promising approaches aimed to inhibit mutant K-RAS are currently under study. Therefore, this review will discuss these approaches and also EGFR therapies, and hopefully, it will draw attention to the need of continued research in the field in order to improve the outcomes in NSCLC patients.

## 1. Introduction

The world health organization (WHO) characterises lung cancer as the second leading cause of death in the world (one in six deaths), being tobacco use the most important risk factor. For both sexes combined, worldwide statistics indicate lung cancer remaining as the leading cause of cancer incidence and cancer mortality (18.4% of the total cancer deaths) and the most commonly diagnosed cancer type (11.6% of the total cases) [1]. Lung cancer arises from the cells of the respiratory epithelium and can be divided into: small cell lung cancer (SCLC), a highly malignant tumour derived from cells exhibiting neuroendocrine characteristics and accounts for 15% of lung cancer cases and non–small cell lung cancer (NSCLC), which corresponds to 85% of cases. NSCLC is further divided into three major pathologic subtypes: adenocarcinoma, squamous cell carcinoma, and large cell carcinoma [2]. The most common subtype of lung cancer is adenocarcinoma comprising approximately 40% of NSCLC cases, followed by squamous-cell carcinoma 25–30% and large cell (undifferentiated) carcinoma 5–10% [3].

**Figure 1:**
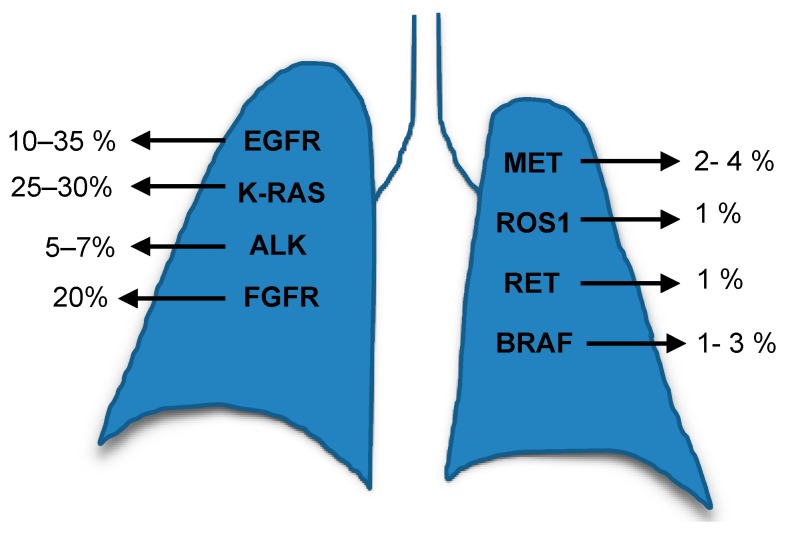
The most frequent altered genes in NSCLC.

The genomic profiling of tumours revolutionised medicine firstly by analysing tumour’s tissues and secondly by the less invasive option, the use of plasma genotyping to detect circulating tumour DNA (ctDNA). These collectively improved the identification of different genetic alterations and also generated options for targeted therapies to support individualised treatment. The alterations frequently found in the main lung cancer subtype, NSCLC, are summarised in Figure 1 [3]. The NSCLC current strategies to develop effective treatments include the study, to a great depth, of different molecular targets such as K-RAS (Kirsten Rat Sarcoma), the epidermal growth factor receptor (EGFR), phosphatidylinositol 3-kinase (PI3Ks), mechanistic target of rapamycin (mTOR), epidermal growth factor receptor 2 (ErbB2), vascular epidermal growth factor receptor (VEGFR), mesenchymal-epithelial transition factor or hepatocyte growth factor receptor (c-MET), anaplastic lymphoma kinase (ALK) and v-Raf murine sarcoma viral oncogene homolog B (BRAF) and others.

The United States Food and Drug Administration (FDA) has approved different drugs designed against the different mutant genes in NSCLC For example, erlotinib, gefitinib, afatinib and osimertinib were developed to target EGFR (discussed later in this review); crizotininb, ceritinib, alectinib and brigatinib were developed to target ALK (crizotinib also targets ROS1). Dabrafenib and trametinib, in combination, were developed to target BRAF V600E [4]. However, the scientific community has had difficulties in pursuing an effective drug against tumours which harbour malfunctioning RAS. In this review, we will focus on the two most affected genes in NSCLC, K-RAS and EGFR, which are also the ones who have attracted a great deal of attention regarding translational research, drug design and clinical trials.

## 2. K-RAS

### 2.1. RAS Biomarkers

**Figure 2:**
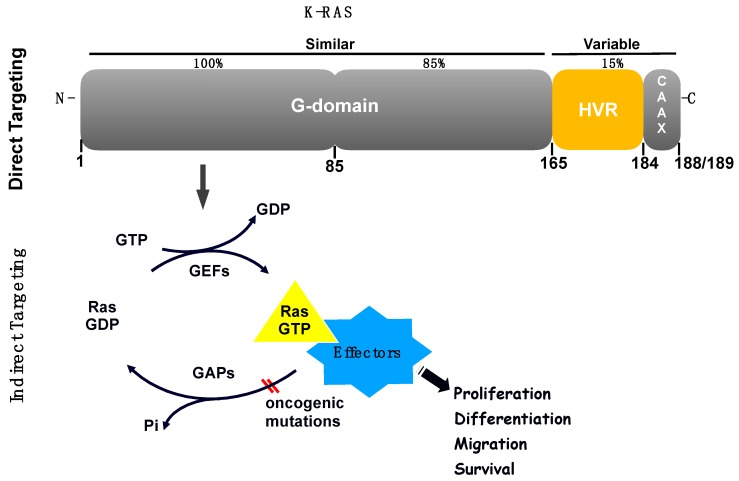
Schematic representation of examples of direct and indirect targeting of K-RAS based on different publications discussed in this review. The G-domain of the K-RAS protein (amino acids 1–165—a highly homologous region among RAS proteins) includes the GTP and GDP-binding pockets of RAS responsible for RAS switch “on” and “off”, respectively. This region is followed by the C-terminus (amino acids 165-188/189), which includes the hypervariable region (HVR) and the CAAX box motif which undergoes posttranslational modifications and determines membrane anchoring (cell location). This region is also the most variable among RAS proteins (15% similarity). K-RAS can be targeted directly on different sites on its own protein structure, or indirectly, via targeting of proteins which interact with K-RAS such as GEFs and GAPs and also K-RAS effectors (located downstream of K-RAS protein).

The RAS family of proteins (H-, K- and N- RAS) share high sequence homology, and are important signalling molecules that regulate cell growth, survival and differentiation by coupling receptor activation to downstream effector pathways. Their structure comprises a highly conserved N-terminal domain responsible for guanine nucleotides binding, and also interaction with activators and effectors, plus a less homologous C-terminal domain responsible for RAS variability and localisation in the cell (Figure 2) [5].

The majority of cancer mutations associated with RAS, are present in the isoform K-RAS at codon 12 followed by codon 13 and 61, leading to a constitutively activated protein [6]. K-RAS mutations have a role in tumour development as well as in tumour progression and resistance occurring frequently in pancreatic, endometrial, colorectal, biliary tract, cervical, and lung cancers. Each different genetic alteration plays a different role in K-RAS-dependent processes. For example, in lung cancer, some mutations were associated with tobacco smoke [7] and, overall, the most frequent alteration occurs at codon 12, the G to T transversions being the most frequent alterations associated with tobacco smoke [7]. Pre-clinical models both in vitro and in vivo were developed to determine the role of K-RAS in tumour progression and response to treatment [8].

Over the past 30 years, no effective anti-RAS inhibitors were accomplished in routine clinical practice rendering RAS as a difficult target. This protein did not appear to present suitable pockets to which drugs could bind [9]. For example, the first attempt was to develop drugs that blocked RAS farnesylation, which led to the clinical development of farnesyltransferase inhibitors. However, the result of clinical trials showed minimal anti-cancer activity [10]. Other strategies were also undertaken but without success. On top of that, lung cancer patients harbouring K-RAS mutations have a poor prognosis due, in part, to the development of resistance to currently available therapeutic interventions. Still, nowadays no effective clinically available therapies exist that directly target the K-RAS oncogene. Furthermore, other alternatives have also attracted attention by focusing on the inhibition of downstream effectors of K-RAS signalling pathways, bypassing the need to target RAS directly. Thus, the different strategies will be discussed in this review.

### 2.2. Examples of Direct Inhibition of RAS

RAS proteins consist of a highly conserved G-domain—a region that undergoes conformational changes between the GDP (guanosine diphosphate)-bound form and the GTP (guanosine triphosphate)-bound form, plus a hypervariable (HVR) C-terminal region that is involved in membrane localisation and it is also the main region that distinguishes the RAS isoforms (Figure 2). The main proposed ways to inhibit RAS have been to develop nucleotide exchange inhibitors, allosteric inhibitors and inhibitors of the RAS-effector interactions [11]. However, the idea to inhibit directly RAS has been hampered for several reasons including the lack of deep hydrophobic pockets in the RAS surface for tight binding of small molecules, the high affinity of RAS towards GTP and GDP and the high intracellular nucleotide concentrations [12].

In terms of allosteric inhibitors, the possibilities of specific targeting mutant K-RAS function without affecting wild-type RAS have been suggested. The common mutations in K-RAS (amino acid residues 12, 61 and rarely on 13) block GAP leading to a constitutive active RAS. The allosteric ligands mainly function by either altering the K-RAS protein structure or disrupting its protein-protein interactions with nucleotide exchange factors, thus favouring its GDP-bound inactive state, preventing downstream signalling. The mutation G12C is the most prevalent in NSCLC and also associated with smoking-related C > A genetic transversions [13]. Nevertheless, it is also frequently found in other types of cancers such as colorectal and pancreatic cancer, suggesting that drugs targeting mutation G12C could be of potential benefit not only in NSCLC treatment but also in other tumours harbouring the same mutation.

The G12C mutation has been targeted directly by electrophilic compounds that bind in a pocket close to G12C, or to the GTP-site itself, and covalently bind to the cysteine residue. The objective of these types of compounds was to lock the RAS protein in its inactive GDP state [14]. Irreversible K-RAS^G12C^ inhibitors block RAS signalling by exclusively binding to and stabilizing the GDP form [14,15,16], and an example is ARS-853 described as a potent inhibitor [16]. Regarding other compounds, tests conducted amongst different cells (including NSCLC cell lines) and patient-derived xenografts, described the structure-based design and identification of a covalent compound called ARS-1620 of promising therapeutic potential. The results showed high potency and selectivity towards K-RAS^G12C^, showing rapid and sustained in vivo target occupancy to induce tumour regression [17]. Nevertheless, another study suggested that ARS-1620 is not always effective as a single agent indicating that signalling adaptation could occur in some instances limiting the efficacy of ARS1620 [18]. In addition, after performing a high-throughput drug screening across 112 drugs in combination with ARS1620, researchers found that combination with PI3K inhibitors could serve as an option to overcome this resistance [18].

Shimomura and colleagues recently investigated molecular targets for K-RAS-activated lung cancer in K-RAS-mutant and wild-type lung cancer cell lines using a drug library of 1271 small molecules, and identified the cytotoxic effects of benzimidazole derivatives on K-RAS-mutant lung cancer cells [19]. In addition, the most recent small-molecule inhibitor termed AMG 510 was presented at the 2019 American Society of Clinical Oncology (ASCO) Annual Meeting indicating its potential to become the first drug to successfully target a K-RAS mutation in patients. The phase I trial tested patients with K-RAS^G12C^-mutantion in different cancer types including NSCLC. The results were partial responses in half of patients with K-RAS^G12C^-mutant NSCLC, and stable disease in most patients with colorectal or appendix cancer [20].

When discussing other K-RAS mutations such as mutation G12D, the aspartate side group was described as a difficult target [21]. Nevertheless, Feng et al. have recently reported that K-Ras^G12D^ has also a potential allosteric small molecule binding site [22]. Their work showed that one compound termed K-RAS allosteric ligand KAL-21404358, when bound to K-RAS^G12D^, impaired its interaction with B-RAF disrupting the RAF-MEK-ERK and PI3K-AKT signalling pathways [22]. 

Other inhibitors such as the case of orthosteric inhibitors were also described. Their binding pockets do not affect the guanosine nucleotide substrate site, instead they can interfere with protein–protein interactions [23]. An example was the design of a cell-permeable synthetic α-helix based on the guanine nucleotide exchange factor SOS which was able to disrupt RAS-SOS interaction resulting in downregulation of Ras signalling in response to receptor tyrosine kinase activation [24]. Molecule inhibitors that block the interaction between K-RAS and its exchange factor SOS1 were also described [24]. Crystal structures of K-RAS G12C–SOS1, SOS1 and SOS2 were obtained to elucidate the binding sites, mode of action, and molecule’s selectivity. The goal was to prevent the formation of the K-RAS–SOS1 complex, resulting in a loading block of K-RAS with GTP, leading to cell proliferation inhibition. The study presented a compound (BAY-293) which selectively inhibited the K-RAS–SOS1 interaction which can be tested in a variety of cancer types including lung cancer [25].

### 2.3. Examples of Indirect Inhibition of RAS

The difficulties in targeting RAS itself stimulated research focused on alternative ways to inhibit its malfunction. Some preclinical and clinical studies have been developed to target RAS downstream pathways (also known as RAS effector pathways, Figure 2), especially the RAF-MEK-ERK and PI3K-AKT pathways which led to several clinical trials. In preclinical studies, the inhibition of both pathways has led to tumour shrinkage representing a hope for clinical testing [26]. In 2018, a phase 1B study evaluated the multitargeted Janus kinase/TANK-binding kinase 1 (TBK1) inhibitor momelotinib combined with the mitogen/extracellular signal-related kinase (MEK)1/MEK2 inhibitor trametinib in patients with platinum-treated, refractory, metastatic, K-RAS-mutant NSCLC. They concluded that the additional use of momelotinib with trametinib did not improve on the activity of single-agent trametinib in K-RAS-mutated NSCLC [27]. Interestingly, trametinib which has been FDA-approved for melanoma, has been tested also in other clinical trials for K-RAS mutant NSCLC patients. A current clinical trial (ClinicalTrials.gov), for example, is testing trametinib in combination with ponatinib, a multityrosine kinase inhibitor (FDA-approved for both chronic myeloid leukemia and Philadelphia chromosome-positive acute lymphoblastic leukemia) therefore indicating that combinational therapies using drugs already approved for other cancer types, could be promising when combined for the treatment in K-RAS mutant NSCLC. Other reviews have nicely addressed different clinical trials based on different pathways and targets affected in lung cancer, such as PI3K, mTOR, BRAF, MEK, MET, HSP90 (Heat shock protein 90) which will not be discussed here [28], in spite that some drugs described in this review could be tested in combination with K-RAS inhibitors or tested in K-RAS mutant patients as combinational therapies.

Mutations in K-RAS can also predict responses to different therapies which do not target K-RAS directly or its downstream pathways. For example, pemetrexed is an anti-metabolite which stops cancer cells making and repairing DNA and it is used alone or in combination with other cancer drugs, such as cisplatin, carboplatin or pembrolizumab, in NSCLC treatment. Despite this, a recent report has suggested that patients with KRAS-mutant lung adenocarcinoma have a poorer outcome on pemetrexed-based first-line chemotherapy [29]. Furthermore, studies using nuclear magnetic resonance (NMR) were performed to identify small molecules able to interact with the RAS-binding domains of proteins downstream of K-RAS as a potential route to block mutant RAS [30].

Immunotherapy has been discussed as a way to treat K-RAS mutant cancer patients. The immune checkpoint inhibitors (ICI) targeting programmed death protein 1 (PD-1) and programmed death ligand 1 (PD-L1) have become a standard treatment option for patients with advanced NSCLC who do not carry targetable mutations, however their efficacy in patients harbouring K-RAS mutations was unknown. Thus, analysis of (PD-L1) expression was performed in a retrospective study including 282 patients. The results showed that in patients with KRAS-mutant NSCLC (all mutational subtypes), the efficacy of ICIs was similar to that for patients with other types of NSCLC. PD-L1 expression appeared more relevant for predicting the efficacy of ICIs in KRAS-mutant NSCLC than in other NSCLC types [31]. Regarding combinational therapy, a clinical trial is currently testing the PD-1 monoclonal antibody termed pembrolizumab in patients with stage IV K-RAS mutant NSCLC in combination with trametinib (ClinicalTrials.gov identifier: NCT03299088).

Over the years, RAS biology research has mainly focused on mutant RAS to develop tools against its oncogenic activity. The importance of wild-type K-RAS allele in various cancers, including lung adenocarcinoma, is still poorly understood. The balance of wild-type and mutant K-RAS in cancer cells could affect the level of signalling pathways and sensitivity to inhibition of these pathways. Interestingly, in 2018, Ambrogio and colleagues, by using a genetically-inducible model of K-RAS loss of heterozygosity, indicated that K-RAS dimerization mediates wild-type KRAS-dependent fitness of human and murine K-RAS mutant lung adenocarcinoma tumour cells and underlies resistance to MEK inhibition [32]. Thus, wild-type K-RAS could play a role to limit the oncogenesis process mainly by connecting the ability of K-RAS to dimerize to the ability of wild-type K-RAS to limit the oncogenic properties of the mutant [32]. K-RAS dimerization impacted MEK inhibitor sensitivity and oncogenic activity of mutant K-RAS [32]. In other cancer types, K-RAS allelic imbalance enhanced fitness and modulated MAP Kinase dependence in cancer [33].

An alternative approach also explored as a way to target K-RAS mutant cancers was synthetic lethality. This method induces cell death by inhibiting the interaction between two co-essential genes rather than inhibiting each one of them alone [34]. Thus, synthetic lethal genes able to interact with the K-RAS oncogene have been searched for and, interestingly, different genes and pathways were found to be required for the survival of cells containing mutant of K-RAS [35]. For example, it was shown that K-RAS mutant NSCLC cells depend on the transcription factor GATA-binding factor 2 (GATA2) [36]. Another proposed idea was to find genes that, when inhibited, cooperate with downstream K-RAS effectors inhibitors, such as MEK inhibitors, to effectively treat K-RAS mutant cancer cells. A shRNA-drug screen strategy identified some of these genes and the anti-apoptotic BH3 family gene BCL-XL (B-cell lymphoma extra-large) appeared as a hit suggesting that a BCL-XL/MEK inhibition could be a potential therapeutic approach for K-RAS mutant cancers [37]. Experiments using ABT-263 (navitoclax), a chemical inhibitor that blocks the ability of BCL-XL to bind and inhibit pro-apoptotic proteins, in combination with a MEK inhibitor, resulted in increased apoptosis in several K-RAS mutant cell lines from different tissue types, including lung [37]. Furthermore, K-RAS mutations in NSCLC were shown to be synthetic lethal alongside CDK4 inhibition [38] and the CDK inhibitor abemaciclib showed antitumor activity in patients with K-RAS-mutant NSCLCs [39]. Recently, a research group showed that loss of the tumour suppressor SMARCA4 was synthetic lethal alongside CDK4/6 inhibition, suggesting that FDA-approved CDK4/6 inhibitors could be effective to treat this subgroup of NSCLC patients regardless of K-RAS status [40]. Figure 2 shows a schematic representation of examples of locations for direct and indirect K-RAS targeting.

## 3. EGFR

### 3.1. EGFR Biomarker

**Figure 3:**
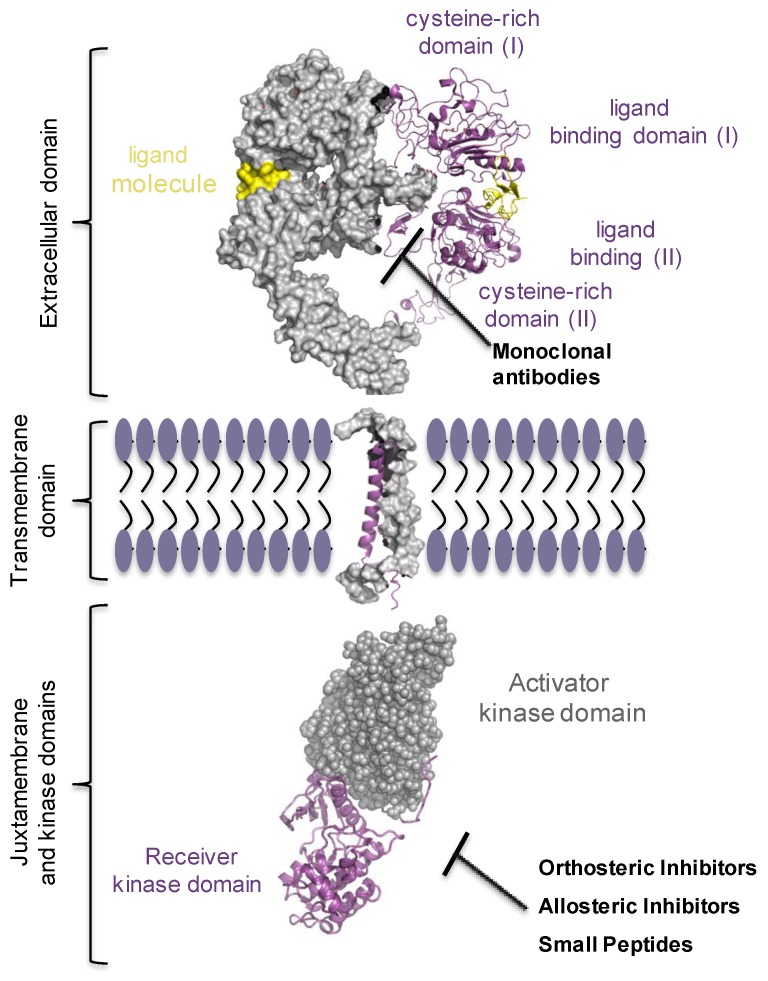
Multimodal approaches targeting oncogenic EGFR in NSCLC. Multiple approaches such as monoclonal antibodies (that target the extracellular ligand-binding domain), orthosteric and allosteric inhibitors, and small peptides designed to block the oncogenic kinase of the EGF receptor, are currently being evaluated in NSCLC therapy. The EGFR crystal structure was drawn using the PyMOL Molecular Graphics System based on protein Data Bank accession codes 3NJP for extracellular and transmembarne domain interfaces, 2M0B for transmembrane domain, and 3GOP for juxtamembrane and kinase domains.

Epithelial growth factor receptor (EGFR/ERBB1/HER1) has been the most studied of the four members of epithelial growth factor receptor family (ErbBs), also including ErbB2/HER2/NEU, ErbB3/HER3, and ErbB4/HER4 receptors [41]. They are all expressed in almost every cell in mammals and convey signals from microenvironment into the cells. Quasi every process in the cell, including proliferation, survival, migration and differentiation, cell and tissue morphogenesis engages ErbB receptor’s signalling [41]. ErbB signalling pathway is frequently found overly active in malignancies of epithelial origin, accounting for 80–90% of all cancer cases, mainly due to gene amplification, point and deletion mutations, or gene fusion, suggesting this pathway as a very attractive target in the oncology field [42,43,44,45,46,47,48,49]. Based on these observations, tailor therapeutic strategies were highly required. Two main possibilities were developed: one was to use small tyrosine kinase inhibitor (TKI) molecules that would target the receptor’s kinase and inhibit the oncogenic form of the receptor [50,51]. The other option was to use monoclonal antibodies (e.g., cetuximab, bevacizumab, nivolumab, pembrolizumab) that target specifically the extracellular ligand-binding domain of the receptor, hence, ceasing the signalling either by increasing the rate of receptor degradation or by preventing its dimerization [52]. Lately, drug programs strategically developed new types of inhibitors, the allosteric inhibitors, hinged on allosteric scenario proposed by Schlessinger’s, Lemmon’s and Kuriyan’s groups, for the EGF receptor. Remarkably, tumour responsiveness and treatment resistance data, matching clinical outcomes, clearly point out the requirement for multimodal approaches to achieve an effective treatment protocol (Figure 3). The ongoing growth of alternative strategies will require to refine the guidelines at which clinical trials may need to shift [53,54,55].

### 3.2. The Complexity of EGF Receptor Signalling in the Cells

The EGF receptor’s signalling inarguably relies on the complex biological network, which starts on the plasma membrane by binding to ligands, soluble growth factors occupying micro-environmental space. Defined by their binding affinity and avidity, ligands impart differences in arrangements of receptor’s homodimer and heterodimer forms, receptor’s phosphorylation, kinetics of receptor’s internalization and endocytic route that cells lately use thoroughly [56,57,58,59,60,61]. Similarly, to all tyrosine kinase (TK) receptors on the plasma membrane, ErbBs are synthesized as a single-pass transmembrane proteins and have an N-terminal extracellular ligand binding module, a single transmembrane helix, a cytoplasmic juxtamembrane segment, intracellular catalytic kinase domain, and a regulatory C-terminus. When bound to a ligand molecule, the receptor’s whole structure is displaced, which immediately follows with the activation of the kinase and specific downstream pathways [61,62,63]. Human EGF-related ligands are soluble polypeptides, made up of less than 100 amino acids. According to their binding affinity, various ligands can be subdivided into three groups; one group specifically binds to EGFR and includes EGF, transforming growth factor α (TGFα), amphiregulin (AR), and epigen (EPG); the second group binds both EGFR and HER4, and includes betacellulin (BTC), heparin binding EGFR (HB-EGF), and epiregulin (EPR); ultimately, a third group consists of all neuregulins (NRGs) 1–4, of which NRG1 and NRG2 bind to both HER3 and HER4 receptors, whereas NRG3 and NRG4 bind only to HER4 [48,64]. Moreover, ligands specify magnitude of the activated downstream signalling pathways, such as RAS/RAF/MEK/ERK mitogen-activated protein kinase (MAPK), phosphoinositide 3-kinase (PI3K)/AKT/mTOR, signal transducers and activators of transcription (STAT) pathway, and phospholipase Cγ [65,66,67]. Upon ligand binding, ErbB receptors go from inactive to active conformations [68,69]. Changes in structural conformation lead to the reorientation of the intracellular kinase positioning two kinases asymmetrically [70,71]. In this model, one kinase, when properly aligned, assumes a role of the ‘activator kinase’ and the C-lobe stabilises the contacts with the N-lobe of the second kinase, termed ‘receiver kinase’. Two kinases could switch the roles, as both receptors become trans-autophosphorylated. When the kinase is activated, it phosphorylates tyrosine residues required to convey signals in the cells [59,72,73,74].

### 3.3. Oncogenic ErbB Variants in NSCLCs and Treatment Approaches

**Figure 4:**
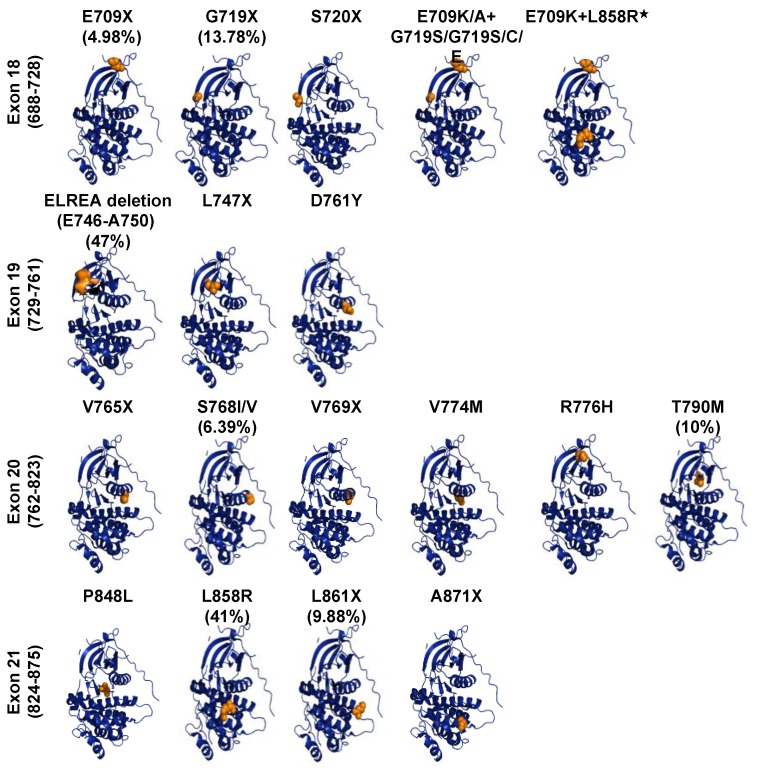
EGFR mutations of kinase domain in NSCLC. Crystal structures of the kinase domain of EGFR are shown. Structures were drown using the PyMole Molecular Graphics System based on the protein Data Bank accession code 4R3P. The EGFR most frequent mutations in NSCLC, highlighted in orange, were mapped on the crystal structure of the EGF receptor’s kinase domain. The frequency of mutations are based on the COSMIC database of somatic mutations.

The concept that EGFR alterations could drive tumour growth established the hypothesis that tyrosine kinase inhibitors could have antitumor effects [75]. Cell culture and transgenic mouse model studies showed that oncogenic alterations in EGFR appeared to have transforming activity [76,77,78,79]. Association of EGFR kinase domain mutations with uncontrolled cell growth, proliferation, and migration have been reported in 32.9% of NSCLC and have proposed EGFR gene as the major oncogenic drug target in lung cancer [45,80,81,82]. Somatic mutations of EGFR found in NSCLC, deposited in the COSMIC database, mainly occur in the exons 18–21 of the kinase domain, the catalytic core of EGF receptor (Figure 4). Uneven distribution of EGFR gene mutations (in-frame, deletions, insertions, duplications and substitutions) are localised or related mostly to the ATP-binding site of EGFR kinase. The most recurrent mutations, which account for 25% of EGFR mutation-positive NSCLCs, are the five exon-19 residues deletion mutation (746ELREA750) that occur in exon19, the β3-αC loop, and the substitution mutation in exon 21 (L858R) [83]. Other gain-of-function EGFR mutations are substitutions at position 719 (G719S/A/C), located within the P-loop of the kinase in exon 18, deletions, insertions and point mutations in exon-19, insertions and point mutations in exon 20 (Ser768Ile, Thr790Met) and point mutations in exon 21 (Leu861Gln) [84,85,86,87,88]. Structural analyses of these mutants revealed that mutations happen in or near the αc-helix, the activation loop, or in the ATP-binding loop. Mutations here disrupt the interactions that detain the kinase in its inactive configuration, shifting the equilibrium towards the active state of the TK [73,87].

The knowledge in structure and biology of the EGF receptor tyrosine kinase resulted in the development of TKIs, which are classified according to their molecular mechanism of action as orthosteric. They are designed to target an active site of the kinase domain and can compete with ATP molecule for binding. When first designed they were classified as the first-generation of tyrosine kinase inhibitors [89] and the FDA has approved two of those in patients with NSCLC termed gefitinib (Iressa) and erlotinib (Tarceva). Clinical data supports the benefit of using tyrosine kinase inhibitors when compared conventional chemotherapy and/or radiotherapy that was conventionally adopted in clinics [90,91,92]. The results comparing the two treatment approaches (chemotherapy *versus* TKIs) were an increase in response rate (from ~56 to 74%) and median survival (from 10 to 14 months) [82,83,91,93,94,95]. Moreover, by analysing the crystal structures of wild-type *versus* mutant EGFRs in complex with kinase inhibitors, it was shown that TKIs preferentially bind the ‘active mutant’ form of the receptor. Direct binding measurement analyses show that gefitinib binds the L858R EGFR mutant form 20-fold more tightly than it binds the wild-type form of the receptor [86,96]. Besides, in vitro analyses show that gefitinib exhibited more affinity for mutant variants, Del747–753 and L858R, than for wild-type EGFR [97]. On the other hand, all the mutant variants were shown not to be equally susceptible to TKIs. Accordingly, heterogeneous effectiveness reflects the structural differences of each inhibitor [98]. For instance, Sliwkowski and his lab showed that L858D is more sensitive than in-frame deletion mutant Del (E746-A750) to erlotinib inhibition [99]. However, in a separate study, patients with NSCLC, harbouring EGFR point mutations (G719X, L858R, L861Q) or deletion 746ELREA750 in exon 19, benefited from either erlotinib/TarcevaTM or gefitinib/IressaTM treatment [82,83,88]. In contrast, several other mutations in exon 20 of the EGFR gene, frequently observed in NSCLC patients, proved to be clinically insensitive to erlotinib or gefitinib. Moreover, these mutations account for nearly 9–11% of all cancers documented with EGFR mutations in NSCLC, representing the third most common type of EGFR mutations, after L858R and exon 19 deletions. Following sequence analyses, mutations in exon 20, happen to be a combination of in-frame insertions and/or duplications of 3–21 base pairs, clustered between 767 and 774 residues, with the most common variant V769_D770insASV. It was found that these mutations reduce the size of the kinase active pocket, and hence inflict insensitivity to erlotinib and gefitinib [100,101,102,103,104,105,106]. In separate studies, through in silico molecular modelling, authors analysed molecular subtype mutations in exon 20 of EGFR and drown a new conclusion: for various insertions in exon 20, the authors anticipated different biological activity with erlotinib treatment [85]. Nonetheless, for the most common EGFR mutations, clinical experience is well established. In contrast, for less common EGFR mutations, which comprised 12.4% of all EGFR mutations, such as amino acid substitutions in E709, G719, S768, and L861 clinical data studies are ongoing. A large cohort study of lung cancer patients reported favourable EGFR TKIs responses in patients who had G719 and L861, however, patients with other rare, uncommon EGFR mutations, failed to respond to kinase inhibitors [98]. Additionally, a rare triple EGFR mutation EGFR-R670W in exon 17 and L833V, and H835L in exon 21, has been described and may respond well to kinase inhibitor treatment [107]. Overall, patients with common mutations in NSCLCs highly respond to first-generation EGFR inhibitors, such as gefitinib and erlotinib, with objective response rates of approximately 70% [82,91].

### 3.4. EGFR Targeting and Drug Resistant Mechanisms in NSCLC

Drug resistance is a well-known phenomenon in cancer therapy and it happens through multiple molecular changes in tumour cells. Tumour growth and survival initially subside upon treatment with TKIs, yet few tumour cells develop resistance mutations inevitably and become resilient to drug therapy [108,109]. Clearly, given therapy becomes inadequate in a particular stage [110]. Tumour growth gradually persists either at the original tumour site or in a distant organ worsen the outcome of the pathology. Darwin’s concept of natural selection applies inarguably to tumour growth and development. It postulates that subclones of cancer cells, owing to the unstable genome, as they undergo incessantly mitosis, acquire the ability to survive in microenvironments when exposed to a drug molecule. This presumably reflects the events developed during the course of treatment in lung cancer, with displayed acquired resistance [111]. Lung cancer’s endogenous tumour defence largely happens due to different reasons such as the altering residues at drug binding sites, ALK and ROS1 rearrangements, and mutations in N-RAS, K-RAS, B-RAF, EGFR and RET [112]. In addition, histological transformation from NSCLC to SCLC is one more described mechanism of the acquired resistance [113].

It is quite puzzling the dynamics and the contributions of the primary mutations in cancer progenitor cells and the acquired mutations evolved in response to drug treatment [95,114,115,116]. Tissue biopsy, regarded as a standard procedure, implies local sampling, and prevents getting enough tumour tissue, therefore, conducts insufficient profiling of tumour genetic aberrant landscape, and, fails to snap the real-time dynamic events that emerge in the course of treatment. When choosing the appropriate therapy for NSCLC patients, it is crucial to obtain an accurate profile of tumour DNA, both “passenger” and “driver” DNA mutations. And one such highly sensitive and less invasive technology for molecular analyses, liquid biopsy, has been recently tested in lung cancer. Profiling circulating tumour cells, cell-free circulating tumour DNA, and non-coding RNA from biological liquids have been proposed, before choosing proper treatment for newly diagnosed patients and drug-resistant NSCLC patients [116,117]. Liquid biopsy indeed offers an advantage to investigate the genetic composition of each patient before treatment decision [118]. Additionally, due to the frequent non-invasive sampling, liquid biopsy allows tracking tumour dynamics, temporal and spatial tumour growth [119]. Therefore, snapping dynamics of tumour growth is the gold standard for decision making in NSCLC treatment, and for this reason, although still challenging for routine use in the clinical setting, liquid biopsy can be seen as a necessary tool in lung cancer therapy.

Regarding EGFR alterations, after 9–11 months of first-line therapy treatment, lung cancer cells acquire gene mutations in a way which often leads to a more increasing and metastatic tumour [120,121]. This hurdle is primarily due to acquired “gatekeeper” mutation T790M in exon 20 of the EGFR gene, which causes resistance in about 50–60% of cases [122,123]. Methionine in position 790 prevents drug binding and increases the affinity for ATP molecules [95,116,121,124,125]. While the EGFR T790M mutation has been linked to the acquisition of resistance to EGFR inhibitors [120,121] and a study reported that families that predisposes for NSCLC could have germline EGFRT790M mutations [126]. In addition to the common EGFRT790M variation, resistance to gefitinib-induced apoptosis is seen in models that contain the novel L747S secondary mutations [127]. Secondary resistant L747S mutation, acquired in cis to activating L858R mutation, attenuated the up-regulation of BIM (also known as BCL2-like 11) and reduced apoptosis [128]. More uncommon mutations are also found, D761Y mutation in exon 19, and T854A in exon 21 of EGFR gene [128,129], substitution of T854 to alanine destabilises the drug contact residues in the EGFR ATP-binding pocket [129]. Furthermore, the study indicated that the inhibitor off-rate impacts on biological drug effects in vivo. An example is given by lapatinib inhibitor, with prolonged downregulation of receptor tyrosine phosphorylation in comparison to gefitinib [130].

In addition to first class inhibitors, the second class was developed, including afatinib, neratinib, and dacomitinib [131]. They were designed to target and irreversibly inactivate EGFR selectively. Cysteine residues, 751 and 773, positioned within the ATP binding pocket of EGFR provided direction for a rational design of a class of EGFR inhibitors that specifically bind with a high-affinity receptor and irreversibly alkylate Cys-773 [132]. Nonetheless, those molecules exhibit biological activity against other receptors of the same family and also structurally related to EGFR [133]. Afatinib molecule was shown to have biological activity towards both wild-type EGFR and mutant EGFRT790M [103,134]. However, it was recently reported that with dose reduction, afatinib could be used as a first-line treatment for tumours harbouring uncommon mutations [135]. Afatinib has currently been approved for first-line treatment of metastatic NSCLC harbouring non-resistant EGFR point mutations (S768I, L861Q, or G719X), exon 19 deletions, and classical EGFR mutations [136,137]. Current evidence indicates that another drug, neratinib, could reduce the risk of recurrence in breast cancer [138], while there are no ongoing clinical trials to assess neratinib in lung cancer [93]. In ongoing clinical trials, with number NCT01774721, patients with advanced NSCLC harbouring one mutation (exon 19 deletion or Leu858Arg) will be assessed with dacomitinib as it has been shown that dacomitinib significantly improved progression-free survival over gefitinib in first-line treatment of patients [139]. Variable degrees of responses to the second-class inhibitors have been found across distinct exon 20 insertions [140]. Low response rates for the second generation of kinase inhibitors (afatinib, dacomitinib and neratinib) have been reported in patients with insertions in exon 20 of EGFR gene [85,104,106,141]. Although these are promising findings, it is important to note that these three inhibitors (afatinib, dacomitinib and neratinib) not only inhibit mutant oncogenic EGFR variants but also wild-type EGFR, and such outcome causes side effects even with the clinically achievable doses [142,143,144,145].

Despite the high response rates (60–70%) and the progression-free survival (9–15 months) achieved with first and second generation of TKIs respectively, patients invariably acquire resistance mutations. Based on these findings, several third-generation EGFR tyrosine kinase inhibitors were developed [100,146,147]. One such example is osimertinib (AZD9291/mereletinib/Tagrisso), an irreversible small-molecule inhibitor of third-class, which covalently binds to cysteine 797 residue in the ATP binding site, and reduced size of the tumour [148,149]. When compared with previous EGFR kinase inhibitors, osimertinib shows significantly less in vitro activity against wild-type EGFR and great efficacy for patients with advanced/metastatic NSCLC who developed EGFRT790M mutation, following progression on first and second classes of TKIs targeting EGFR. In the mouse model of EGFR-mutated NSCLC, osimertinib had antitumor activity in tumours driven by EGFRL858R mutation comparable to that of afatinib, but it was more effective than afatinib in EGFR double mutants, L858R/T790M or exon 19 del/T790M EGFR [125,148]. Similarly, and due to distinct structure, osimertinib can be used for EGFR 19 deletion, L858R, and exon 20 insertion mutations as first-line EGFR-TKI [150,151,152]. Recently, the FLAURA study has shown significant progression-free survival benefit and prolongation of all post-progression outcome endpoint adopting osimertinib in the first-line setting for EGFR-mutated NSCLC [153]. After analysing 13 randomized controlled trials, including data from 3539 patients with EGFR-mutated NSCLC, Holleman et al. showed favourable efficacy of osimertinib in terms of PFS and OS compared to all other EGFR-TKIs [154]. Relative to first-line treatment with EGFR TKIs (erlotinib and gefitinib), osimertinib significantly prolongs median progression-free survival (PFS) [155]. Recently, more mutant-selective molecules of third-generation have been developed, including rociletinib (CO-1686), olmutinib (HM61713), nazartinib (EGF816), mavelertinib (PF-7775), YH5448, avitinib (AC0010), naquotinib (ASP8273) and WZ4002. They were all designed to specifically target EGFR-activating mutant EGFRT790M and refrain from having an effect on wild-type EGFR [145,152,156]. Nazartinib inhibits a panel of patient-derived cell lines that have a deletion in exon 19, L858R/T790M, deletion in exon19/T790M, or three exon 20 insertion mutations [157]. Rociletinib (CO-1686), sharing common structural features with osimertinib, promotes tumour regression in tumour xenograft and transgenic models, and is currently being evaluated in phase I/II clinical trials in EGFR-mutant NSCLC [145]. While WZ-4002 has shown much promise, it did not progress into clinical trials. Nonetheless, despite the emerging success and recognised advantages of treatment therapy with osimertinib, some drawbacks appeared to develop. The emergency of subclones harbouring EGFRC797S somatic gene mutation is one example. Mutant variant C797S prevents receptor binding to osimertinib inhibitor. Similarly, mutant variants L792H and G796R circumvent the inhibitory effect of osimertinib. Of the acquired mutations in the ATP binding sites, T790M-L858R only affect the rate of binding to irreversible inhibitors and do not affect the extent of inhibition [158]. Interestingly, datasets derived from structural studies indicated that EGFR is also an allosteric enzyme, and it hence appeared to be an ideal target for allosteric inhibitors. Suppressing reorientation of the symmetric inactive dimer to form asymmetric active dimer kinase activation, presents new opportunities for optimal tumour inhibitory function. Mechanistically, the allosteric inhibitor should hold back two kinase domains to interact with each other. For instance, research recently published by Liang et al. suggested that phosphorylated EGFR dimer alone is not sufficient to activate RAS signalling, highlighting that, in the context of ligand stimulation, the conformational changes induced by ligands is a crucial determinant for the efficient signal propagation of SOS-RAS-MAPK pathway [159]. When compared to other inhibitors, the allosteric modulator compounds bond surfaces with no functional domain, and hence evolutionarily less conserved, anticipating the advantage to be highly specific. As a proof-of-concept, a dimer-inhibiting compound was developed, the NSC56452. Such a compound inhibits receptor autophosphorylation and cell proliferation in HeLa cells by disrupting allosteric activation of EGF-stimulated dimer [160]. The action is likely EGFR specific due to its interaction with the ectodomain of the EGF receptor. Another attractive example of this new class of inhibitors is EAI045 inhibitor [161]. This compound inhibits T790M/L858R mutant EGFR forms, with low-nanomolar potency in biochemical assays. Interestingly, Jia et al. observed that treatment combination with cetuximab, a therapeutic monoclonal antibody that blocks EGFR dimerization, renders kinase susceptible to EAI045 inhibitor [157]. In mouse models of lung cancer driven by L858R/T790M and L858R/T790M/C797S EGFR mutations, the tumour shrinks when treated synergistically with EAI045 and cetuximab, suggesting that EAI045 and cetuximab exhibit mechanistic synergy. Authors also speculated that combining an allosteric inhibitor with ATP-site-directed compound could be used to prevent treatment-associated resistance mutations in the receptor itself. Treatment with osimertinib and JBJ-04-125-2, a mutant-selective EGFR allosteric inhibitor, increases apoptosis, a more efficient inhibition of cellular growth, and increased efficacy both in vitro and in vivo [162].

An EGFR kinase domain H-helix analogue peptide, termed EHBI2, which successfully inhibits EGFR activation and signalling, was recently proposed. This could represent a new strategy for EGFR targeting [163] adding on the numbers of drug combinations that could be applied in clinical practice. Monitoring signs of treatment resistance via molecular data is crucial to achieve the best benefit for NSCLC patients. About 50% of cases, who developed drug resistance in NSCLCs, happen to be due to inherent cancer cell heterogenic background, modifications of expression levels, a permutation of signalling pathways, epithelial-mesenchymal transition (EMT), SCLC transformation, or PI3K mutations [113]. For instance, the permutation of compensatory/parallel signalling, named bypass track resistance, results with either activation of the receptors that belong to same receptor family of ErbBs, or, instead, other kinase receptor families, like MET receptor or IGF receptor [101,129].

Combinational therapy is widely required for synergistic antitumor activity [164]. Reactivation of ErbB3, a critical activator of PI3K, was found to tighten therapy treatment with gefitinib. In a mouse model of lung cancer driven by EGFR T790M/L858R mutant, treatment with an antibody MM-121, which blocks ligand-induced activation of ErbB3 and cetuximab, induced durable tumour regression [165]. On the other hand, osimertinib, when used in combination with pemetrexed or cisplatin delayed development of resistance mechanism [166]. In 12% of NSCLCs that lack the resistant second-site EGFRT790M mutation, acquired resistance is due to amplification of HER2, as revealed by FISH analyses. Clinical experience and experiments in vitro suggest that co-targeting ErbB2, with afatinib, and EGFR with afatinib and cetuximab have a far more superior benefit [167]. Equally preventing shedding of heregulin, thereby inhibiting ErbB3 signalling, correlates with gefitinib sensitivity [168].

Activation of downstream signalling pathways, including RAS/MAPK kinase and PI3K/AKT/mTOR signalling, are observed in patients with developed, acquired resistance to antibodies or TKIs, via gene mutations/amplifications or release of feedback loops. Treatment design to target the PI3K signalling pathway, promoting cell survival, metabolism, and proliferation, and the MEK kinase pathway, tested in the preclinical model, showed some promising activity. Patients with EGFR mutations in lung cancer, resistant to erlotinib alone were more sensitive to the combination of EGFR and MEK inhibitors. Combined treatments with EGFR kinase inhibitor, and WZ4002, MEK inhibitor, or knocking down ERK2 suppressed cell growth [169]. Similarly, the PI3K/AKT/mTOR pathway also plays a role in acquired resistance to EGFR-targeted therapy [170]. Phosphorylated AKT was observed in a large proportion of NSCLC patients (50–73%) and was associated with poor prognosis [170]. Loss or reduced expression of PTEN is present in about 70% of NSCLC, followed by mutations in PI3KCA (2–5%) and AKT1 (1–2%). It is well established that cancer disease often relapses at distant sites due to the formation of metastatic cancer cells. The signalling process by which the epithelia transit to mesenchymal (EMT) is often activated in a fraction of treated NSCLCs. The selective pharmacological inhibitor of EMT transcription factor, TWIST1, was shown to inhibit growth, and induce apoptosis [171]. Another report by Shimamura et al. has shown that the oncogenic EGFR requires Hsp90 for proper conformation folding. Therefore, Hsp90 inhibitors, such as geldanamycin, may represent a novel strategy to adopt in NSCLCs treatments [172]. Detecting genetic variants, when disease initiates and during the treatment therapy, are needed in order to contract cancer growth [87]. It is important to note that if there are no local drugs available for mutation switch, chemotherapy treatment might be considered [119,173]. Overcoming drug resistance is critical in order to achieve the best benefit for NSCLC patients, therefore understanding the molecular biology behind the disease is fundamental.

**Figure 5:**
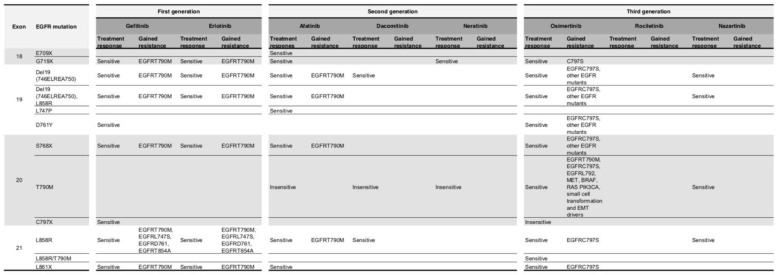
EGFR mutant status and drug resistance mechanisms.

A summary table (Figure 5) for EGFR mutant status and drug resistance mechanisms is shown.

### 3.5. EGFR and K-RAS Concomitant Mutations

NSCLC patients harbouring EGFR mutations might simultaneously present alterations in other genes, which could represent concomitant driver mutations. Although tumours harbouring co-occurring mutations are unusual, they exist and represent a rare molecular subtype, which might affect response to different treatments. Thus, some studies were performed in order to evaluate this. For example, Next-generation sequencing (NGS) for concomitant driver mutations was performed on *EGFR*-mutated tumour samples from erlotinib-treated patients and the result showed that most concomitant mutations (including EGFR/K-RAS) did not impact the response to first-line erlotinib-treatment [174]. Furthermore, a recent retrospective study testing 3774 samples from NSCLC patients (tested for EGFR, ALK, ROS1, K-RAS and BRAF) observed that only 1.7% harboured mutations in two or three genes, and among these patients, EGFR/K-RAS was the most frequent coalteration (31.7%), followed by ALK/K-RAS (17.5%) [175]. In addition, they concluded that patients harbouring coalterations tend to benefit more from TKI therapy than from chemotherapy [175]. In contrast, another report suggested that a subgroup of EGFR mutant tumours with concomitant driver mutations affected the activity of first-line EGFR TKIs [176]. They showed that the progression free survival (PFS) was 11.3 months *versus* 7 months in patients without and with other mutations, respectively (log-rank test univariate: *p* = 0.047) [176]. These different findings suggest that more studies, from different populations around the world, are necessary to further confirm the real role of concomitant mutations in NSCLC.

## 4. Conclusions

Inarguably K-RAS and EGFR have been the focus of numerous studies which have tried to improve several aspects in lung cancer biology. The phenomenon of drug resistance observed in clinics is one of the most critical challenges that the oncologists have to deal with, and, to achieve the best benefit for NSCLC patients, a solution is still needed. Additionally, finding a specific drug to target K-RAS has not been an easy journey. That is why research scientists, structural biologists, chemists, and clinicians scrutinize successes and failures of cancer drug technology continuously, putting forward new resolutions and strategies, inciting pharmaceutical industry to design and develop drugs with new criteria, including the development of direct and indirect methods to inhibit targets. Tumour stratification has been discussed as important for the success of different therapies by precisely targeting selected patients to ensure its efficacy and decrease side effects. In addition, early diagnosis makes possible to achieve long-lasting remission and the analysis of free circulating tumour DNA is a promising tool to achieve this goal.

## Abbreviations

K-RAS	Kirsten Rat Sarcoma
EGFR	Epidermal growth factor receptor
mTOR	mechanistic Target Of Rapamycin
ErbB2	Epidermal Growth Factor Receptor 2
VEGFR	Vascular Epidermal Growth Factor Receptor
PI3Ks	Phosphatidylinositol 3-kinase
ALK	Anaplastic Lymphoma Kinase
BRAF	v-Raf murine sarcoma viral oncogene homolog B
SCLC	Small Cell Lung Cancer
NSCLC	Non–small Cell Lung Cancer
WHO	World Health Organization
ctDNA	circulating tumour DNA
c-MET	Mesenchymal-epithelial transition factor
GDP	Guanosine diphosphate
GTP	Guanosine triphosphate
TBK1	TANK-binding kinase 1
MEK	mitogen/extracellular signal-related kinase
FDA	Food and Drug Administration
HSP90	Heat shock protein 90
GATA2	GATA-binding factor 2
BCL-XL	B-cell lymphoma extra-large
TKI	Tyrosine kinase inhibitor
TGFα	Transforming growth factor α
AR	Amphiregulin
EPG	Epigen
BTC	Betacellulin
HB-EGF	Heparin binding EGFR
EPR	Epiregulin
NRG	Neuregulins
N-RAS	Neuroblastoma RAS viral oncogene Homolog
H-RAS	Harvey RAS viral oncogene Homolog
PFS	Progression-free survival
MAPK	Mitogen-activated protein kinase
ICI	Immune checkpoint inhibitors
PD-1	Programmed death protein 1
PD-L1	Programmed death ligand 1
